# Effect of Polyvinylpolypyrrolidone Surfactant on Characteristics of Iron-Oxide Nanoparticles Synthesized by Using Recycled Waste Permanent Magnets

**DOI:** 10.3390/mi13112020

**Published:** 2022-11-19

**Authors:** Sung-Jei Hong, Ajin Jo, Sang Hyeok Hong, Byeong Jun Kim, Young Sung Kim, Suwon Yang, Jae-Yong Lee

**Affiliations:** 1 Korea Electronics Technology Institute, Seongnam 13509, Republic of Korea; 2 Nano IT Convergence Engineering, Graduate School of NIDE Fusing Technology, Seoul National University of Science & Technology, Seoul 01811, Republic of Korea; 3 Han Chung RF, Co., Ltd., Incheon 21678, Republic of Korea

**Keywords:** iron-oxide nanoparticles, permanent magnet scraps, recycling, polyvinylpolypyrrolidone (PVP), crystal structures, particle size, morphology, magnetic properties

## Abstract

In this study, iron oxide nanoparticles (FeO_x_ NPs) were synthesized by using Fe solution recycled from NdFeB permanent magnet scrap. Furthermore, the effect of polyvinylpolypyrrolidone (PVP) as a surfactant on the characteristics of the FeO_x_ NPs was investigated. Firstly, Fe solution was prepared by using 10% H_2_SO_4_ solution and Na_2_SO_4_ salt. In addition, three reducing agent solutions were prepared by dissolving PVP in 0.5 M NH_4_OH solution in distilled (D.I.) water with concentrations of 0 wt%, 1 wt%, and 2 wt%, respectively. Each reducing agent solution was added dropwise into the Fe solution to precipitate three precursors of FeO_x_ NPs, and they were heat-treated at 400 °C to prepare three FeO_x_ NPs samples, P0, P1, and P2. In X-ray diffractometer (XRD) analysis, diffraction peaks of P0 sample are consistent with the Fe_3_O_4_ with (311) preferred orientation. The XRD peak shifted from Fe_3_O_4_ to Fe_2_O_3_ structure as PVP concentration increased, and the crystal structure of P2 sample was transformed to Fe_2_O_3_ with (104) preferred orientation. Brunauer, Emmett, and Teller (BET) specific surface area increased in proportional to PVP concentration. HRTEM observation also supported the tendency; the particle size of the P0 sample was less than 40 nm, and particle size decreased as PVP concentration increased, leading to the particle size of the P2 sample being less than 20 nm in width. In addition, particle morphology started to be transformed from particle to rod shape as PVP concentration increased and, in the P2 sample, all the morphology of particles was transformed to a rod shape. Magnetic properties analysis revealed that the P0 sample exhibited the highest value of magnetic moment, 65.6 emu/g, and the magnetic moment was lowered in the P1 sample, and the P2 sample exhibited the lowest value of magnetic moment, 2.4 emu/g.

## 1. Introduction

Recently, NdFeB permanent magnets have been widely used in component parts requiring high magnetic magnets such as motors and computers of home appliances, electric vehicles and hybrid vehicles, A/V components, magnetic separators, aerospace systems, and other equipment [1]. As the markets related to the NdFeB permanent magnets grow, their scraps are also gradually increasing. Scraps generated from those products must be recycled in an eco-friendly manner for sustainable growth of related industries [2,3]. Recycling of the permanent magnet scraps mainly consists of rare earth elements such as neodymium (Nd) and dysprosium (Dy) contained in the magnets. The scraps contain not only rare earth elements such as Nd and Dy but also a large amount of Fe elements [4]. There are several papers that have been published on the synthesis of iron oxide nanoparticles (FeO_x_ NPs) from waste like coal fly ash to incense sticks ash [5,6,7,8,9,10,11,12,13,14,15]. Although there have been many studies on the recycling of Fe as well as the rare earth elements, the recovery of Fe in the NdFeB has not been paid an intensive amount of attention. Moreover, from the NdFeB scraps, the materialization of the recycled Fe to synthesize FeO_x_ NPs has not been widely reported yet. For example, Fe_2_O_3_ is the most stable FeO_x_, has a high resistance to corrosion, and is low cost, biocompatible, eco-friendly and non-toxic. In addition, it shows n-type semiconducting properties (E_g_ = 2.1 eV) under ambient conditions [16]. It has been investigated extensively due to its wide commercial and industrial applications in many fields such as pigment, catalyst, sensor, environmental pollutant cleanup agent, electrode material, biomedical material, and magnetic material [17,18,19,20]. In addition, Fe_3_O_4_ NPs have attracted the extensive attention of many researchers because of their superparamagnetic properties, great magnetic activity, large surface area, low cost, and good capacitance [21,22,23]. The surface of Fe_3_O_4_ NPs can undergo rapid, reversible redox reaction, exhibiting strong pseudocapacitive behavior [24] and, therefore, Fe_3_O_4_ NPs are considered as a very favorable electrode material. In addition, Fe_3_O_4_ NPs have been the most promising and popular candidate of contrast-enhancing probes for magnetic resonance imaging (MRI) due to their proven excellence of biocompatibility, low cost, and low toxicity [25]. It is expected that the high added value FeO_x_ NPs will be made if the recycled Fe element is materialized to the oxide NPs as well as decreasing carbon emissions. Unfortunately, study on the FeO_x_ NPs from NdFeB magnet scraps has been rarely reported. Recently, we have experienced that FeO_x_ NPs was synthesized from NdFeB magnet scraps [26]. With the amount of the NdFeB permanent magnet scraps projected to increase rapidly in the near future, research on efficient recycling of Fe elements from the scraps to synthesize high value-added FeOx NPs is demanded in order to decrease waste discharge from the magnet. Therefore, in this study, FeO_x_ NPs were synthesized by using the Fe element recycled from NdFeB permanent magnet scrap, and the characteristics, i.e., physical and magnetic properties of the FeO_x_ NPs, were observed. Furthermore, polyvinylpolypyrrolidone (PVP) as a surfactant was added when they were synthesized, and the effect of the PVP on their characteristics was investigated.

## 2. Materials and Methods

### 2.1. Preparation of FeO_x_ NPs

**Figure 1:**
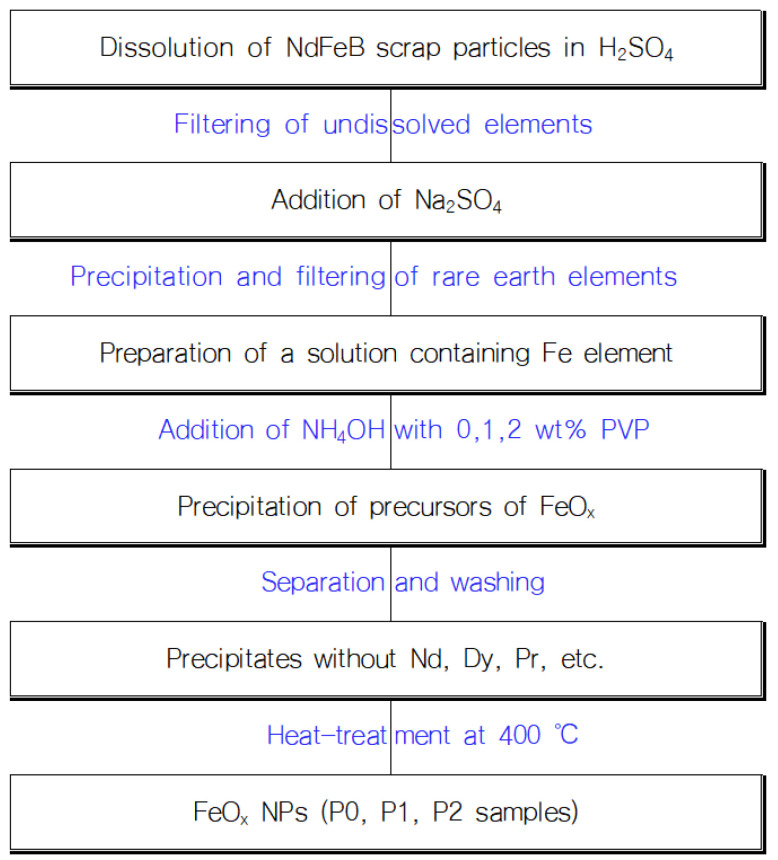
Schematic representation of the procedure and mechanism for the formation of FeO_x_ NPs.

As a raw material, Fe solution was prepared by using the following process; in addition, 10 g of NdFeB scrap particles were added to 100 mL of a 10% sulfuric acid (H_2_SO_4_, 95%, Daesung Science Co., Siheung, Republic of Korea) solution, and the solution was filtered to separate the undissolved outer deposit elements. Then, sodium sulfate (Na_2_SO_4_, 99.0%, OCI Company Ltd., Seoul, Korea) salt was added to the filtered solution, and the rare earth elements contained in the solution were precipitated to separate. As a reducing agent, ammonia (NH_4_OH, ACS reagent, 28.0–30.0% NH_3_ basis, Sigma Aldrich, St. Louis, MO, USA) solutions were prepared by diluting it into distilled (D.I.) water to a concentration of 0.5 M, followed by dissolution of 0 wt%, 1 wt%, and 2 wt% of PVP (PVP10, average mol wt 10,000, Sigma Aldrich, St. Louis, MO, USA) into the solution [27], respectively. After that, the Fe solution was diluted with D.I. water to a concentration of 10 wt%, and each reducing agent solution was added dropwise into the Fe solution to precipitate three precursors of FeO_x_ NPs. The precipitates were then centrifugated and dried at 80 °C in a convection oven. The precipitates were washed several times to separate residual rare earth elements such as Nd. To determine heat-treatment temperatures where components are thermally decomposed, except Fe to be oxidized, examination of thermal behavior of the precipitates was conducted using thermogravimetric analysis (TGA) and differential thermal analysis (DTA) at temperatures ranging from 25 °C to 550 °C with a rising rate of 10 °C/min. Based on the result, the heat-treatment temperature was determined from the thermally decomposed point, 400 °C, and three FeO_x_ NP samples were prepared by heat-treatment at that temperature. We named the samples as P0, P1, and P2, respectively. A schematic representation of the procedure and mechanism for the formation of FeO_x_ is shown in Figure 1.

### 2.2. Characterizations of FeOx NPs

The prepared FeO_x_ NP samples were characterized as follows: impurities in the FeO_x_ (P2) sample were analyzed with an ICP Emission Spectrometer (iCAP 6000 Series, Thermo Scientific, Cambridge, UK). Crystal structures were analyzed by using a X-ray diffractometer (XRD, Empyrean, Malvern Panalytical B.V., Malvern, UK) with Cu K radiation over a range of 2θ angles from 20° to 80°. In addition, their surface areas were measured with Brunauer, Emmett, and Teller (BET) surface area analyzer (Tristar II 3020, Micromeritics, Norcross, GA, USA). Their particle size and morphology were observed by using by a high resolution transmission electron microscope (HRTEM, JEM-ARM200F, JEOL, Tokyo, Japan). Saturation magnetization of samples was measured by using a vibrating sample magnetometer (VersaLab VSM, Quantum Design, Inc., San Diego, CA, USA) under room temperature and applied magnetic fields of up to 30,000 Oe.

## 3. Results

### 3.1. Thermal Behaviors

**Figure 2:**
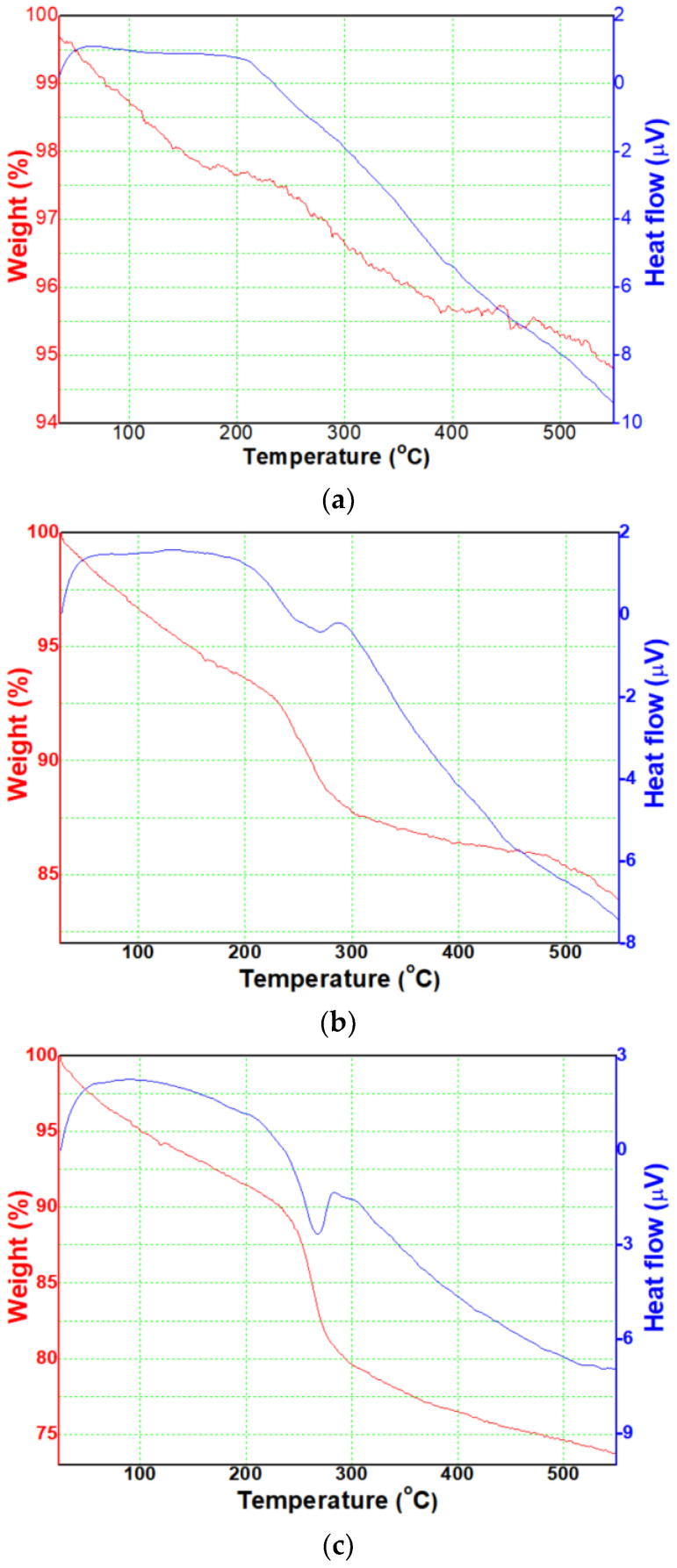
Thermal behaviors of the precipitates with (**a**) 0 wt%; (**b**) 1 wt%; (**c**) 2 wt%.

Firstly, we analyzed thermal behaviors of the three precipitates. As a result of TGA, in Figure 2a, precipitate without PVP showed that the weight (red graph) linearly decreased as the temperature increased up to c.a. 400 °C and remained c.a. 94.5% above that temperature. The DTA result (blue graph) showed a wide peak ranging from 250 °C to 350 °C with a central peak at c.a. 300 °C, and it is supposed that the precursor is thermally decomposed at around the temperature. In Figure 2b, precipitate with 1 wt% PVP showed that the weight (red graph) slowly decreased with increasing temperature and, especially, it rapidly decreased ranging from 240 °C to 300 °C. Above that temperature, it slowly decreased again and remained c.a. 84% at 550 °C. The DTA result (blue graph) showed peaks ranging from 270 °C to 310 °C with the central peak at c.a. 290 °C, and the peak range is narrower than that of the sample in Figure 2a. In Figure 2c, precipitate with 2 wt% PVP showed that the weight (red graph) slowly decreased with increasing temperature and, especially, it rapidly decreased ranging from 240 °C to 300 °C with a higher slope than that of the sample in Figure 2b. Above that temperature, it slowly decreased again and remained c.a. 74% at 550 °C. The DTA result (blue graph) showed a similar tendency to that of the sample in Figure 2b, and it is supposed that the precursor is thermally decomposed at around the temperature.

From those results, it was known that the FeO_x_ precursor, which is reduced and precipitated from the iron byproduct solution extracted from the permanent magnet scrap, is expected to be able to synthesize crystalline FeO_x_ NPs above 400 °C by thermal decomposition of the residue component in the precursor. Thus, heat-treatment temperature was determined as 400 °C, and the three precipitates were heat-treated at the temperture, etc. In addition, as shown in Table 1, it was analyzed that rare earth elements, such as Nd, Dy, and Pr, were not found after heat-treatment of the P2 sample, one of the synthesized FeO*_x_* NPs samples. It is attributed that the residaul rare earth elements were separated from the precipitates through the washing process. Therefore, it is expected that the residual rare earth elements were not contained in P0, P1 samples.

### 3.2. Crystal Structure

**Figure 3:**
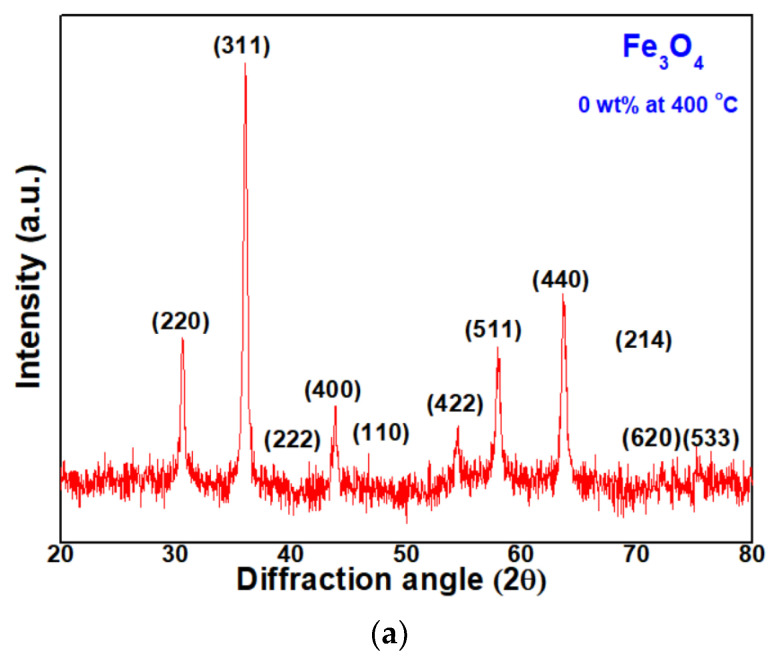

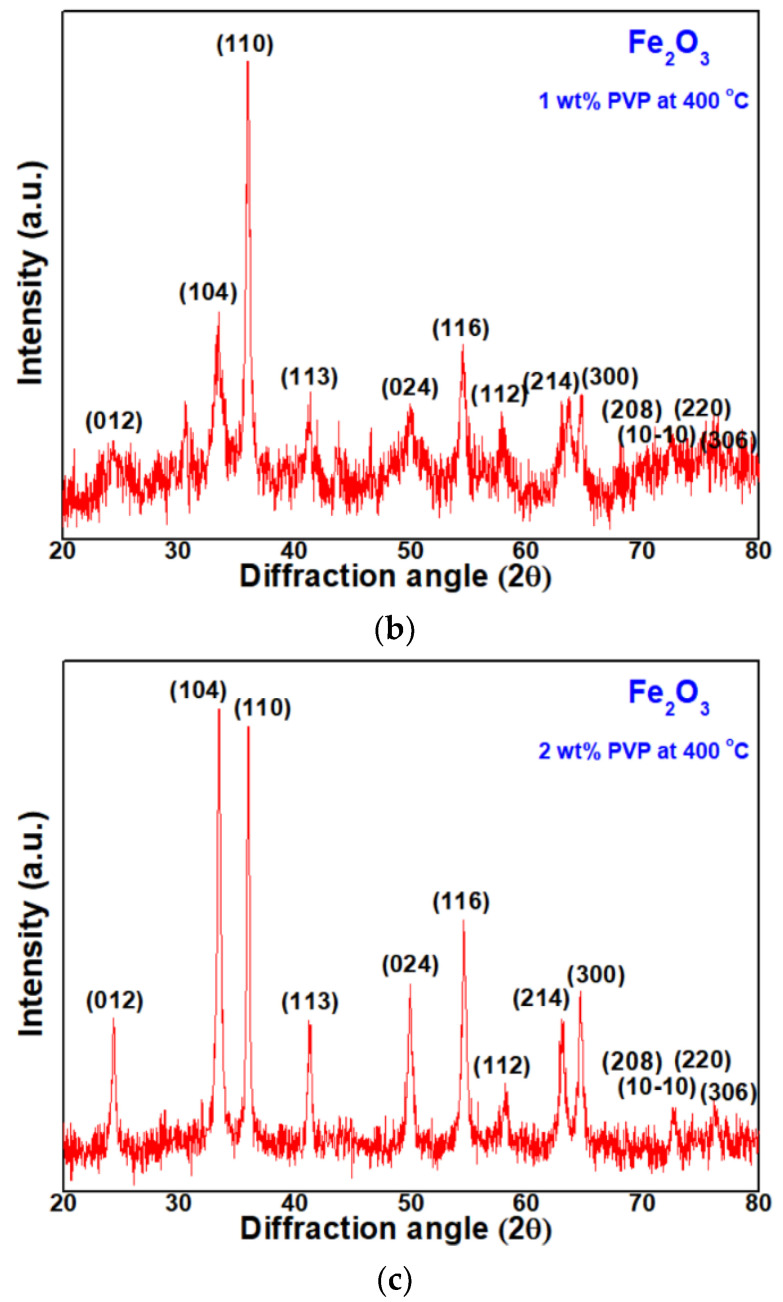
X-ray diffractometer (XRD) patterns of FeO_x_ NPs with (**a**) 0 wt%; (**b**) 1 wt%; (**c**) 2 wt% polyvinylpolypyrrolidone (PVP).

Then, here, the authors analyzed the crystal structure of P0, P1, and P2 samples heat-treated at 400 °C. Their XRD patterns are shown in Figure 3a–c. In Figure 3a, the P0 sample has its main diffraction peak at 36.00°, which is corresponding to <311> directions, i.e., the growth was the largest in that direction, indicating a Fe_3_O_4_ structure with the <311> preferred orientation [28]. In Figure 3b, the P1 sample has its main diffraction peak at 35.94°, which corresponds to the <110> direction. It seems an intermediate phase changing from Fe_3_O_4_ structure to Fe_2_O_3_ structure growing towards the <104> preferred orientation [29]. The FeO_x_ NPs with 2 wt% PVP, in Figure 3c, has the main diffraction peak at 33.47°, which corresponds to <104> directions. It is a stable Fe_2_O_3_ structure growing towards the <104> preferred orientation, i.e., hematite structure [30].

### 3.3. Particle Size and Morphology

**Figure 4:**
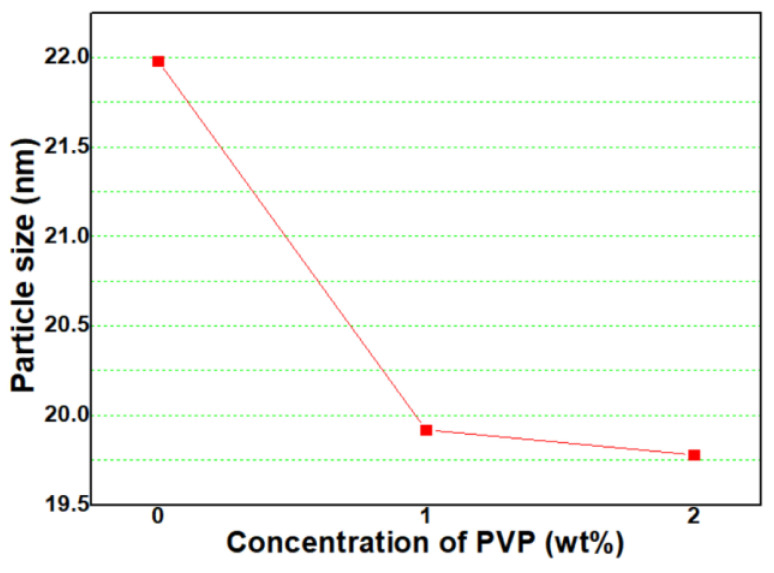
Variation of particle size (calculated by using Equation (1)) of FeO_x_ NPs with concentration of PVP.

From the XRD peaks, the full-width-half-maximum (FWHM) of each of the main peaks, (311) at P0, (110) at P1, and (104) at P2 samples, was different with concentrations of PVP. It is supposed that the size of the FeO_x_ NPs was affected by the addition of the PVP. From the XRD peaks, particle size can be calculated by using Scherrer’s equation [31] as

t = 0.9 λ/B cos θ_B_ (1)

where t, λ, B, and θ_B_ are particle size, wavelength (0.1542 nm for CuK_α_ radiation), full-width-half-maximum (FWHM) of a peak in radians, and diffracted angle, respectively. In Equation (6), the peak intensity increases along with a reduction in the peak half width, indicating the growth of FeO_x_ NPs. Thus, as the FWHM is widened, the particle size is smaller. In Figure 4, calculated with Equation (1), particle sizes of P0, P1, and P2 samples were 22.0 nm, 19.9 nm, and 19.5 nm, respectively. Particle size steeply decreased from that of the P0 sample when 1 wt% PVP was added, and the particle size slightly decreased from that of the P1 sample when 2 wt% PVP was added.

**Figure 5:**
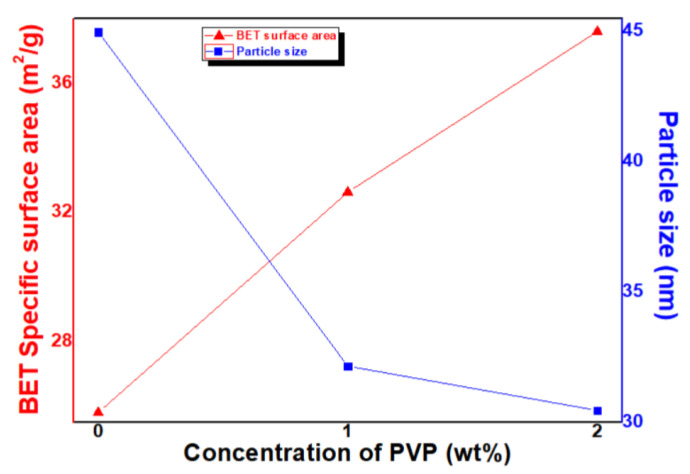
Variation of Brunauer, Emmett, and Teller (BET) specific surface area (SSA) and particle size (calculated by using Equation (2)) of FeO_x_ NPs with a concentration of PVP.

Then, BET specific surface area (SSA) of the samples were measured, and the results are shown in Figure 5. The SSA of P0, P1, and P2 samples are 25.80 m^2^/g, 32.62 m^2^/g, and 37.59 m^2^/g, respectively.

The particle size was calculated from the BET SSA as follows (see Equation (2)):

D = 6/ρ·d(2)

where D, ρ, and d is particle size, SSA, and density (5.17 g/cm^3^ for Fe_3_O_4_ and 5.24 g/cm^3^ for Fe_2_O_3_), respectively. In Figure 4, the calculated particle sizes of P0, P1, and P2 samples are 44.98 nm, 32.15 nm, and 30.46 nm, respectively. The SSA tends to increase with a rising concentration of PVP. In addition, in the conversion to particle size, the particle size decreases with a rising concentration of PVP.

**Figure 6:**
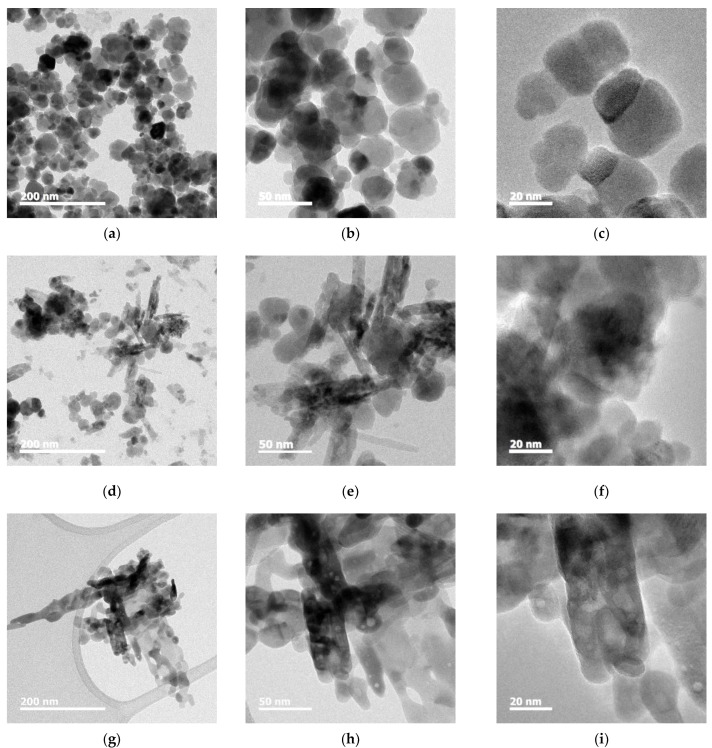
HRTEM observations of FeO_x_ NPs with (**a**–**c**) 0 wt%; (**d**–**f**) 1 wt%; (**g**–**i**) 2 wt%.

For certifying the particle sizes of FeO_x_ NPs with different concentrations of PVP, the samples were observed with HRTEM. In the case of the P0 sample, shown in Figure 6a–c, sphere-shaped Fe_3_O_4_ NPs with particle sizes of c.a. ~40 nm are observed, and some of the NPs are agglomerated. When 1 wt% PVP was added to FeO_x_ NPs (P1 sample), shown in Figure 6d–f, particle size of Fe_2_O_3_ NPs decreased to c.a. ~35 nm, and some of the rod-shaped NPs were observed. In addition, in the case of 2 wt% PVP addition (P2 sample), shown in Figure 6g–i, most of the NPs were transformed to rod-shaped NPs with a width of c.a. ~20 nm.

### 3.4. Magnetic Properties

**Figure 7:**
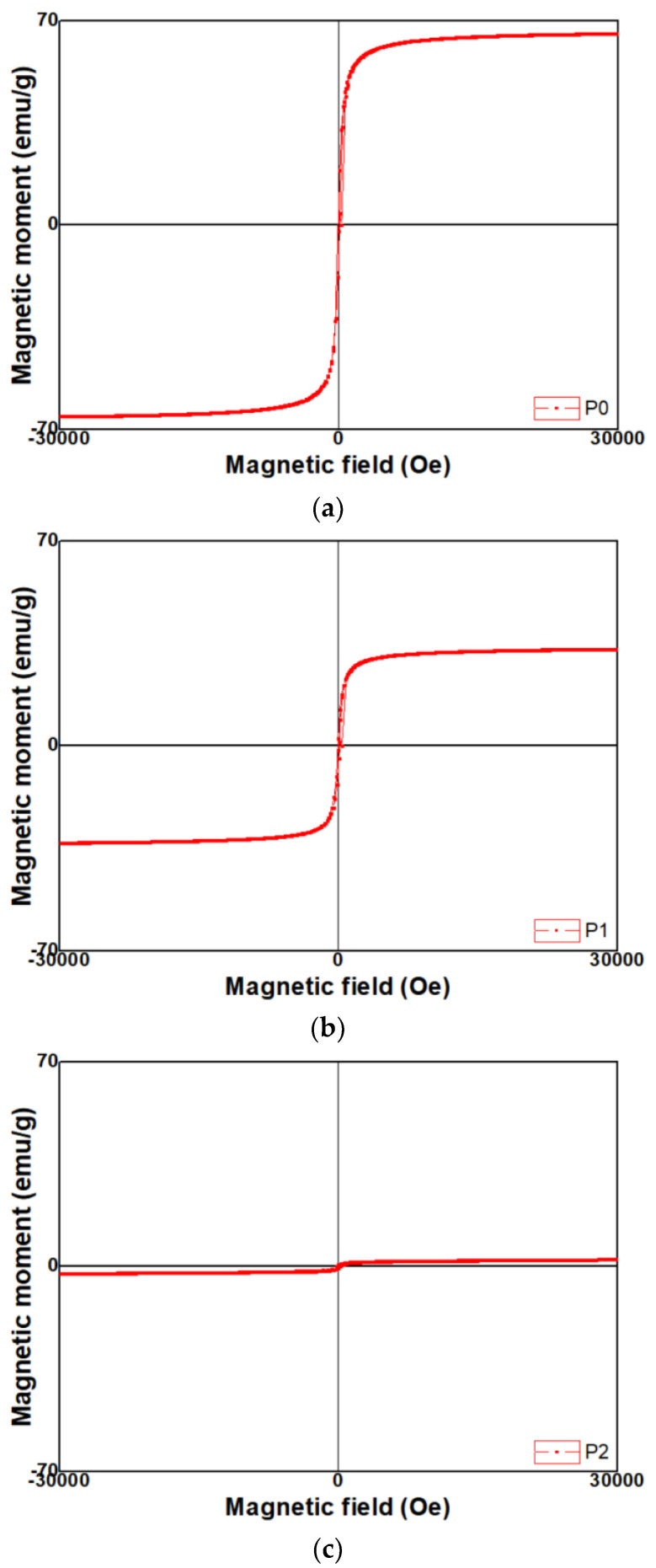
Magnetic properties of FeO_x_ NPs with (**a**) 0 wt%; (**b**) 1 wt%; (**c**) 2 wt% PVP.

Magnetic hysteresis curves of FeO_x_ NPs evaluated with VSM at room temperature were presented in Figure 7a–c. All the samples have symmetric hysteresis and saturation magnetization. In addition, the difference in magnetic properties was found depending upon the FeO_x_ NPs samples. When a magnetic field was applied to 30,000 Oe, magnetic moments of P0, P1, and P2 were measured as 65.6 emu/g, 33.1 emu/g, and 2.4 emu/g, respectively. The P0 sample exhibited the highest value of magnetic moment, whereas the magnetic moment decreased in the P1 sample, and the P2 sample exhibited the lowest value of magnetic moment. Meanwhile, coercivity of P0, P1, and P2 was measured as 75 Oe, 51 Oe, and 150 Oe, respectively. The P2 sample exhibited the highest value of coercivity, whereas coercivity decreased in the P0 sample, and the P1 sample exhibited the lowest value of coercivity.

## 4. Discussion

### 4.1. Crystal Structure

In this study, the magnetite (Fe_3_O_4_) crystal structure was formed by reducing the Fe element in the scrap solution with alkaline NH_4_OH solution as a reducing agent in which PVP is not contained. It is reported [32] that, in aqueous solution, Fe^2+^ and Fe^3+^ ions are co-precipitated in an alkaline medium such as NH_4_OH. In this procedure, hydroxides of ferrous and ferric ions, ferrous hydroxide (Fe(OH)_2_), and goethite (α-FeOOH) are coprecipitated as precursors in an alkaline solution. By setting a molar ratio of ferrous/ferric equal to 0.5, the solid phase reaction between hydroxides results in the formation of Fe_3_O_4_.

In the case of the P0 sample, the peaks are indexed to those of a magnetite [28,32], i.e., it appears to be reduced and precipitated in the form of Fe(OH)_2_ and Fe(OH)_3_ as follows:

FeSO_4_ + Fe_2_(SO_4_)_3_ + 8NH_4_OH → Fe(OH)_2_ + 2Fe(OH)_3_ + 4((NH_4_) _2_SO_4_) (3)

In addition, after heat-treatment, it is supposed that the precipitates, Fe(OH)_2_ and Fe(OH)_3_, are crystallized to Fe_3_O_4_ as follows [32]:

Fe(OH)_2_ + 2Fe(OH)_3_ → Fe_3_O_4_ + 4H_2_O↑ (4)

In addition, it is supposed that H^+^ ions are removed out of the (OH)^-^ ions attached to the Fe^3+^ ions by adding PVP. When enough H^+^ ions are removed, a neutral complex precipitate, hydrated iron (III) oxide (FeOOH), is formed as follows [21]:

Fe_3_O_4_ + 0.25O_2_ + 4.5H_2_O → Fe(OH)_3_ (5)

Fe(OH)_3_ → FeOOH + H_2_O (6)

In addition, FeOOH is crystallized to Fe_2_O_3_ by oxidizing Fe^2+^ ions to Fe^3+^ ions with O^2-^ ions as follows:

2FeOOH → Fe_2_O_3_ + H_2_O (7)

Thus, it was known that crystal structure of the FeO_x_ NPs was affected by the concentration of PVP.

### 4.2. Particle Size and Morphoogy

Similar to the particle sizes calculated from BET SSA (Equation (2)), it is clearly observed that the particle size decreases as the concentration of PVP increases. However, it is found that there is a little difference in conversion particle size values between XRD FWHM and BET SSA. It is attributed that this difference is owing to measurement principles [33]. In the case of XRD FWHM, X-rays can penetrate through the crystal size to provide information; therefore, the calculation of size is not related to the particles but to the crystals. In addition, it is difficult to provide separation and distinguishing of broadening through the crystallite size from the broadening due to other parameters and factors. However, in the case of BET SSA, under normal atmospheric pressure and at the boiling temperature of liquid nitrogen, the amount of nitrogen adsorbed in the relationship with pressure gives the SSA of NPs. Errors always exist and successful calculation methods are those which can decrease the errors in the best possible way to yield more accurate data.

The variation in the particle size by concentration of PVP is attributed to improvement of agglomeration during growth driven by the surface additives surrounding the particles. One of mechanisms to increase the nanoparticle size is owing to mainly particle surface migration [34]. According to the transformation kinetics,

D = exp(-Q/kT)(8)

where D, Q, k, and T are mean particle size, activation energy for particle surface migration, Boltzmann constant, and heat-treatment temperature, respectively. When the activation energy for particle growth of FeO_x_ NPs becomes high by the surface additive, PVP, the growth of the NPs is suppressed under the constant heat-treatment temperature leading to lowering of the particle size.

In addition, it is supposed that rod shaped Fe_2_O_3_ by the addition of PVP is owing to hydrothermal growth. It is reported that the rod morphology is based on the anisotropic crystal growth mechanism of the lenticular Fe_2_O_3_ nanorods [35]. Growth of Fe_2_O_3_ nanorods is considered as a two-stage process: (1) the growth and dissolution of intermediate FeOOH nanostructures, alongside precipitation of Fe_2_O_3_ NPs; and (2) the agglomeration and coarsening of Fe_2_O_3_ NPs into Fe_2_O_3_ nanorods. Dissolution of the FeOOH phase leads to the release of Fe^3+^ anions back into the solution to supply the growth of Fe_2_O_3_ NPs, which in turn coalesced to form lenticular Fe_2_O_3_ nanorods. In addition, strong surfactant absorption on mediating the lenticular shape of Fe_2_O_3_ crystal surfaces stabilized the primary Fe_2_O_3_ NPs size during the growth. A decrease in total surface energy by the surfactant is the driving force for the crystal growth kinetics of FeOOH and Fe_2_O_3_.

Thus, it is known that the control of the particle size and shape is controlled by addition of a surfactant, PVP.

### 4.3. Magnetic Properties

As particle size of the P0 sample is larger than that of P1 and P2 samples, the magnetic moment of the P0 sample is much higher than that of P1 and P2 samples. That is, as the particle size of FeO_x_ NPs increases, the saturation magnetization also increases in proportion to the particle size. This phenomenon may be attributed to the small particle size effect since a noncollinear spin arrangement occurs primarily at or near the surface, which results in the reduction of magnetic moment in FeO_x_ NPs [36,37,38]. Moreover, it is reported that saturation magnetization decreases with smaller size due to surface spin effects [9,39,40]. In the literature, the saturation magnetization of bulk Fe_3_O_4_ is 92 emu/g, and a decrease of magnetization arises from the disordered surface spins, whereas there is perfect A-O-B superexchange interation resulting in ferrimagnetism at the interior of the particle. Judging from those things, it is known that particle size of FeO_x_ NPs affects their magnetic properties.

In addition, it seems that the tendency of the magnetic properties of the samples is also dependent on their crystal structure. As analyzed, the P0 sample has Fe_3_O_4_ (magnetite) crystal structure, whereas P1 and P2 samples have Fe_2_O_3_ ones, and, especially, (104) direction is preferentially oriented in the P2 sample, indicating that it is the hematite phase. In connection with these results, it seems that their magnetic properties are dominated by their crystal structure as well as particle size. In the case of the P0 sample, magnetic hysteresis curve (Figure 6a) exhibits ferrimagnetic behavior [41]. However, the magnetic hysteresis curve of the P1 sample (Figure 6b) exhibits ferromagnetic behavior [42]. Moreover, the magnetic hysteresis curve of the P2 sample (Figure 6c) exhibits weakened ferromagnetic behavior as the crystal structure grows to a (104) direction, leading to the hematite phase [30,38]. In addition, despite the smallest particle size of the P2 sample, its coercivity exhibited the highest value, whereas coercivity of the P0 sample exhibited a middle level, despite its particle size being higher than that of two samples. It is reported that the coercivity decreases when particle size is reduced [40]. In this case, it seems that the crystal structure factor dominates the magnetic properties rather than the particle size factor. That is, their magnetic properties were determined not by particle size but by crystal structure. Therefore, it is known that the crystal structure of FeO_x_ NPs dominantly affected their magnetic properties.

## 5. Conclusions

In this study, FeO_x_ NPs were well synthesized by using a solution containing an Fe element recycled from NdFeB permanent magnet scrap. In addition, their physical and magnetic properties were intensively affected by PVP surfactant when they were synthesized with 0.5 M NH_4_OH reducing solution followed by heat-treatment at 400 °C. FeO_x_ without PVP has XRD peaks consistent with the Fe_3_O_4_ crystal structure with (311) preferred orientation, and the XRD peak shifted from Fe_3_O_4_ to Fe_2_O_3_ structure as PVP concentration increased leading to Fe_2_O_3_ crystal structure with (104) preferred orientation when 2 wt% PVP were added. In addition, the BET specific surface area increased in proportion to PVP concentration. HRTEM observation also supported the tendency; the particle size of Fe_3_O_4_ NPs without PVP was less than 40 nm, and the particle size decreased as PVP concentration increased, so that the particle size of Fe_2_O_3_ NPs with 2 wt% PVP was less than 20 nm in width. In addition, particle morphology was changed from particle to rod-shape as PVP concentration increased and, in Fe_2_O_3_ NPs with 2 wt% PVP, all the morphology of the particle was changed to rod-shape. Magnetic properties analysis revealed that Fe_3_O_4_ NPs without PVP exhibited the highest value of magnetic moment, 65.6 emu/g. The magnetic moment was lowered with an increase of PVP concentration, and Fe_2_O_3_ NPs with 2 wt% PVP exhibited the lowest value of magnetic moment, 2.4 emu/g.

## Figures and Tables

**Table 1:** Impurities in the FeO_x_ (P2) sample analyzed with ICP.

Elements	Concentration (PPM)
Al	83
Ca	296
Ce	0
Dy	0
Fe	643,200
La	0
Nd	0
Pr	0
Si	0
Sm	0
Y	0
Na	433
S	105
Mn	472
P	64

## Data Availability

Not applicable.
